# Fragmented Ventricular Complexes and Blood Pressure Variability Assessed by Ambulatory Blood Pressure Monitoring in Patients With Metabolic Syndrome

**DOI:** 10.7759/cureus.59950

**Published:** 2024-05-09

**Authors:** Ajay Mishra, Fakhare Alam, Saboor Mateen, Firdaus Jabeen, Mehvish Anjum, Neel Mamrawala

**Affiliations:** 1 Internal Medicine, Era’s Lucknow Medical College and Hospital, Lucknow, IND; 2 Internal Medicine, All India Institute of Medical Sciences, Gorakhpur, Gorakhpur, IND; 3 Internal Medicine, Era's Lucknow Medical College and Hospital, Lucknow, IND

**Keywords:** ecg (electrocardiogram), metabolic syndrome, hypertension, ambulatory blood pressure monitoring, fragmented qrs

## Abstract

Introduction

Hypertension is a leading risk factor for the development of cardiovascular and metabolic derangements. In patients with metabolic syndrome (MetS), hypertension is one of the cornerstones showing high variability which is detected in ambulatory blood pressure monitoring. Fragmented ventricular complexes on ECG are seen as hypertensives and are a viable and easy measure of myocardial fibrosis even in the absence of obvious hypertrophy.

Aim

The present study was undertaken to study the blood pressure variability in patients of MetS with fragmented QRS (fQRS) versus normal ventricular complexes (QRS).

Results

Out of 100 patients, 22 (22%) had fQRS complexes. Hypertension and diabetes were the most prevalent associated in both groups but a difference was seen with coronary artery disease, which was significantly associated in the fQRS group (8.97% vs 95.45%, p<0.001) as compared to the non-fQRS group. Significant differences were observed in waist circumference (p=0.019), triglyceride (p=0.006) and left ventricular ejection fraction (p<0.001) between the two groups. There was a marked difference (p<0.05) between heart rate variability during day and night time between normal and fQRS sub-groups, being higher in the latter. A similar pattern of change was observed for systolic and diastolic blood pressures and associated dipping.

Conclusion

Significant differences exist between heart rate and blood pressure changes in patients with fQRS of MetS, thus making fQRS a potent indicator of cardiovascular status.

## Introduction

Blood pressure (BP) is characterised by marked short-term fluctuations such as beat-to-beat, minute-to-minute, hour-to-hour, and day-to-night changes. Twenty-four-hour ambulatory BP monitoring (ABPM) is the most widely available tool to assess short-term BP variability (BPV), its patterns, underlying mechanisms and clinical implications [1]. A large and increasing number of studies support the evidence that BPV represents a strong and independent risk factor for cardiovascular diseases (CVDs) [2,3], as well as for hypertension-related morbidity and mortality [4].

Metabolic syndrome (MetS) is a cluster of disorders that puts an individual at risk of developing serious health issues. MetS has been defined by multiple criteria. The two widely used criteria for identifying MetS are the National Cholesterol Education Programme (NCEP) Adult Treatment Panel (ATP-III) and the International Diabetic Federation (IDF). NCEP-AT III criteria recognise the presence of any three of the conditions as MetS, while IDF emphasises the presence of central obesity with any two other conditions as MetS [5,6]. Various studies have indicated that among individuals with MetS, of the five risk factor components, hypertension was observed most frequently in both genders (up to as high as 85%) [7,8].

Mere recording of a BP reading at the clinic to diagnose or predict the outcome of a cardiovascular problem is no longer accepted. Repeat measurements are needed to ensure the accuracy of these measurements. Circadian variability affects BP and heart rate changes [9], thereby cementing it as one of the markers of hypertension as well as influencing endocrine disorders [10], renal disorders [11], headache and neuropathic pain [12], behavioural patterns [13] and most importantly in metabolic and cardiovascular disorders [14,15].

Twenty-four-hour ABPM is the most widely available tool to assess short-term BPV and its wide use in the practice and research of hypertension allows meaningful insight into BPV’s patterns, clinical significance, and underlying mechanisms. It is well-accepted that short-term (within 24 hours) variations are affected by sympathetic activation, peripheral resistance (caused by arterial elastic properties) [16], blood viscosity, vasoconstrictors effects, and emotional and behavioural factors.

Fragmented QRS (fQRS) was defined by an additional R wave (R') or notching within the QRS complex. fQRS on 12-lead electrocardiography (ECG) was originally defined as narrow QRS complex duration (<120 ms) and improved identification of prior myocardial infarction in patients who are being evaluated for coronary artery disease (CAD) [17]. A narrow fQRS complex as a ventricular conduction abnormality is a sign of myocardial fibrosis and is associated with adverse outcomes in various CVDs [18,19]. Therefore, the presence of fQRS on ECG, as an indicator of myocardial fibrosis, may be a sign of increased BP even in the absence of left ventricular (LV) hypertrophy.

The present study aimed to study the BPV in patients of MetS with fQRS and normal QRS.

## Materials and methods

The present research was carried out as a prospective observational study in a tertiary care North Indian hospital over a span of 24 months from 2021 to 2023. Outdoor and hospitalized patients with MetS who met the inclusion and exclusion criteria were enrolled in the study.

Inclusion and exclusion criteria

All patients above the age of 18 years, of either gender, after informed consent diagnosed with MetS by NCEP ATP-III criteria [5] were included in the study. Patients not meeting the inclusion criteria with either bundle branch block, non-sinus rhythm/arrhythmia, stable angina pectoris or acute coronary syndrome, valvular heart disease and those with malignancy on radiotherapy were excluded.

Sample size

The sample size of the study was 100 patients.

Methodology

All the diagnosed cases of MetS fulfilling the inclusion criteria were first clinically examined. During the examination, personal details, duration of MetS, family history, and details of co-morbidities were collected. Patients were referred for ECG and 2D ECHO, and on the basis of the findings of the above tests; patients were divided into two groups: fQRS and non-fQRS (normal QRS). Blood samples were collected and BP monitoring was done on ABPM machines.

Procedure for ABPM

ABPM was performed using a mobil-o-graph r (IEM, Aachen, Germany), and the results were analysed using an HMS Client-Sever-4.0 system (Hypertension Management Software, IEM, Stolberg, Germany). The BP and heart rate were monitored for one whole day: every 30 min during the day (0700 to 2200 hours) and every 60 min during the night (2200 to 0700 hours). More than 90% of the readings were valid.

Observed parameters

The observed parameters for ABPM included peak day-time heart rate, peak night-time heart rate, average night-time heart rate, average day-time heart rate, minimum day-time heart rate, minimum night-time heart rate, diastolic and systolic BP, average BPV and BP circadian rhythm.

Statistical analysis

The statistical analysis was done using Statistical Package for the Social Sciences (IBM SPSS Statistics for Windows, IBM Corp., Version 21, Armonk, NY). The values were represented in number (%) and mean±SD. Chi-square and student ’t’ tests were used and a p-value of <0.05 was deemed significant.

## Results

Of the total 100 patients of MetS, 22 patients (22%) have fQRS complex. The mean age in patients with a normal QRS complex was 58.51±9.19 years whereas that with fQRS had a mean age of 51.55±8.58 years which was statistically significant (p-value 0.002) (Table 1).

**Table 1:** Age distribution between patients of metabolic syndrome with and with our fragmented QRS

Variable	Normal (n=78)		Fragmented QRS (n=22)		t	p-value
	Mean	±SD	Mean	±SD		
Age (years)	58.51	9.19	51.55	8.58	3.19	0.002

The percentage of males and females was 58.97% and 41.03% in normal and 72.73% and 27.27% in fQRS ECG. On the basis of gender, both groups were comparable. In terms of co-morbidities prevalence of hypertension, type 2 diabetes mellitus (T2DM), and coronary heart disease (CHD) were 82.05%, 84.62%, and 8.97% in normal and 100.00%, 100.00%, and 95.45% in fQRS ECG, respectively. On the basis of hypertension and T2DM, both groups were comparable, whereas CHD was significantly more common in the fQRS ECG. Of the 78 patients, a total of 64 (82.05%) had angiotensin-converting enzyme/angiotensin receptor blockers (ACEI/ARB) usage in the normal ECG and 22 (100%) patients in the fQRS group. On the basis of ACEI/ARB, the two groups were comparable (Table 2).

**Table 2:** Comparison and summation of variables between normal and fragmented QRS ECGs T2DM: type 2 diabetes mellitus; CAD: coronary artery disease; ACEI: angiotensin-converting enzyme (ACE) inhibitors; ARB: angiotensin receptor blockers

Variables	Normal (n=78)		Fragmented QRS (n=22)		Chi sq.	p-value
	n	%	n	%		
Gender						
Male	46	58.97	16	72.73	0.86	0.355
Female	32	41.03	6	27.27		
Co-morbidities						
Hypertension	64	82.05	22	100.00	3.22	0.073
T2DM	66	84.62	22	100.00	2.53	0.112
CAD	7	8.97	20	95.45	54.36	<0.001
One co-morbidity	73	93.59	22	100.00	0.442	0.51
Two co-morbidity	56	71.79	22	100.00	6.38	0.01
Three co-morbidities	25	32.05	20	90.91	21.7	<0.001
ACEI/ARB Usage						
Yes	64	82.05	22	100.00	3.22	0.073
No	14	17.95	0	0.00		

The mean BMI and waist circumference were 29.19±3.05 and 91.60±7.47 in normal and 29.29±2.33 and 87.39±6.73 in fQRS ECG, respectively. Although BMI was not significantly different but mean waist circumference was significantly different between the two groups. The mean haemoglobin (Hb), total leukocyte count (TLC) (x103) and platelet were 12.06±1.90, 8.24±3.14 and 2.01±0.73 in normal and 12.86±1.78, 7.90±2.19 and 2.12±0.77 in fQRS ECG, respectively. In addition, the mean Hb, TLC (x103) and platelet were not significantly different between normal and fQRS ECG. Blood sugars (both fasting and post-prandial), glycated Hb and renal profile were only comparable. With respect to lipid profile, only serum triglycerides were significantly different between the two groups (Table 3).

**Table 3:** BMI and blood profile difference between the two study groups BMI: body mass index; VLDL: very low-density lipoprotein; LDL: low-density lipoprotein; HDL: high-density lipoprotein; HbA1c: glycated haemoglobin

Variables	Normal Range	Value	Standard Deviation	Value	Standard Deviation	Chi sq.	p-value
BMI and Waist Circumference							
BMI (kg/m^2^)	18.5 to 24.9	29.19	3.05	29.29	2.33	-0.14	0.886
Waist circumference (cm)	Males ≤94cm; Females ≤80 cm	91.60	7.47	87.39	6.73	2.38	0.019
Haematological Profile							
Haemoglobin	Males: 14-18 g/dL; Females: 12-16 g/dL	12.06	1.90	12.86	1.78	-1.78	0.078
Total leukocyte Count	4.5 to 11.0 × 10^9^/L	8.24	3.14	7.90	2.19	0.48	0.635
Platelets	1.5-4.0 10^3^/µL	2.01	0.73	2.12	0.77	-0.65	0.516
Biochemical Profile							
Serum Cholesterol	<200 mg/dL	203.50	56.47	185.45	43.23	1.39	0.169
Serum Triglyceride	<150 mg/dL	180.14	46.64	219.05	85.19	-2.82	0.006
VLDL	2 to 30 mg/dL	45.59	16.96	51.27	15.71	-1.41	0.162
LDL	<100 mg/dL	78.08	35.69	69.45	15.59	1.10	0.274
HDL	Males <40 mg/dL; Female <50 mg/dL	35.46	10.33	38.64	7.90	-1.33	0.185
Urea	5 to 20 mg/dL	40.41	22.02	38.50	37.78	0.30	0.763
Serum Creatinine	0.7 to 1.3 mg/dL	0.99	0.50	0.89	0.14	0.99	0.326
Others (Individual values are taken)							
Blood Sugar (BS) Fasting	Diabetes: ≥126 mg/dL	170.64	36.42	183.27	28.46	-1.50	0.137
Blood Sugar Post-prandial (PP)	Diabetes: ≥200 mg/dL	237.35	56.89	232.73	39.82	0.36	0.722
HbA1C	Diabetes ≥6.5%	8.17	1.96	7.83	1.16	0.77	0.445

Table 4 shows the comparison of the mean heart rate (HR) between normal and fQRS ECG. The mean HR Peak DT (Day Time), HR Peak NT (Night Time), HR Av (Average) DT, HR Av NT, HR Min DT and HR Min NT were 94.15±20.27, 88.96±17.76, 83.17±17.47, 80.01± 16.00, 79.92±22.35 and 78.22±21.68 in the normal ECG group and 107.32±15.91, 95.45±15.32, 94.64±12.76, 90.18±11.93, 85.09±13.99 and 88.45±17.95 in the fQRS ECG. The mean HR Peak DT, HR Av DT, HR Av NT and HR Min (Minimum) NT were significantly higher in the fQRS ECG than in the normal ECG. The HR Peak NT and HR Min DT were also higher in fQRS, but not significantly different.

**Table 4:** Comparison of mean heart rate between normal and fragmented QRS ECG HR: heart rate; DT: day time; AV: average; NT: night time; Min: minimum

	Normal (n=78)		Fragmented QRS (n=22)		t	p-value
	Mean	±SD	Mean	±SD		
HR Peak DT	94.15	20.27	107.32	15.91	-2.81	0.006
HR Peak NT	88.96	17.76	95.45	15.32	-1.56	0.122
HR Av DT	83.17	17.47	94.64	12.76	-2.87	0.005
HR Av NT	80.01	16.00	90.18	11.93	-2.77	0.007
HR Min DT	79.92	22.35	85.09	13.99	-1.03	0.307
HR Min NT	78.22	21.68	88.45	17.95	-2.03	0.046

The mean systolic blood pressure (SBP) between normal and fQRS ECG. The mean SBP Peak DT, SBP Peak NT, SBP Av DT, SBP Av NT, SBP Min DT and SBP Min NT were 150.33±12.66, 135.04±10.76, 127.08±9.82, 117.93±9.16, 126.08±15.45 and 118.94±14.52 in the normal ECG group and 171.82±23.45, 148.86±15.55, 141.18±16.72, 126.09±9.96, 127.45±23.92 and 126.59±15.20 in the fQRS ECG. The mean SBP Peak DT, SBP Peak NT, SBP Av DT, SBP Av NT, and SBP Min NT were significantly higher in the fQRS ECG than in the normal ECG. The SBPMin DT were also higher in fQRS, but not significantly different (Table 5). The mean diastolic blood pressure (DBP) Peak DT, DBP Peak NT, DBP Av DT, DBP Av NT, DBP Min DT and DBP Min NT were 96.03±8.71, 86.42±8.51, 83.53±8.57, 79.44±9.56, 77.27±8.22 and 72.40±8.56 in the normal ECG group and 103.91±8.39, 90.64±6.02, 93.00±8.46, 90.91±9.87, 91.45±12.46 and 79.59±9.97 in the fQRS ECG. The DBP Peak DT, DBP Peak NT, DBP Av DT, DBP Av NT, DBP Min DT and DBP Min NT were significantly higher in the fQRS ECG than in the normal ECG (Table 5).

**Table 5:** Comparison of mean systolic and diastolic blood pressure variability and their average during daytime (DT) and night time (NT) between normal and fragmented QRS ECG SBP: systolic blood pressure; DBP: diastolic blood pressure; AV: average; DT: day time; NT: night time; Min: minimum

	Normal (n=78)		Fragmented QRS (n=22)		t	p-value
	Mean	±SD	Mean	±SD		
SBP Peak DT	150.33	12.66	171.82	23.45	-5.70	0.000
SBP Peak NT	135.04	10.76	148.86	15.55	-4.79	0.000
SBP Av DT	127.08	9.82	141.18	16.72	-5.02	0.000
SBP Av NT	117.93	9.16	126.09	9.96	-3.61	0.000
SBP Min DT	126.08	15.45	127.45	23.92	-0.32	0.747
SBP Min NT	118.94	14.52	126.59	15.20	-2.16	0.033
DBP Peak DT	96.03	8.71	103.91	8.39	-3.78	0.000
DBP Peak NT	86.42	8.51	90.64	6.02	-2.17	0.032
DBP Av DT	83.53	8.57	93.00	8.46	-4.59	0.000
DBP Av NT	79.44	9.56	90.91	9.87	-4.94	0.000
DBP Min DT	77.27	8.22	91.45	12.46	-6.32	0.000
DBP Min NT	72.40	8.56	79.59	9.97	-3.36	0.001

Table 6 shows the comparison of mean arterial pressure (MAP) between normal and fQRS ECG. The mean MAP (Max) and MAP (Min) were 119.78±12.35, 74.37±13.59 in normal and 119.23±13.62 and 75.91±15.58 in fQRS ECG. In addition, the mean MAP (Max) and MAP (Min) did not differ significantly between normal and fQRS ECG. In addition, the mean diurnal variation (S) did not differ significantly between normal and fQRS ECG.

**Table 6:** Comparison of mean MAP and mean heart rate between normal and fragmented QRS ECG MAP: mean arterial pressure; Max: maximum; min: minimum

	Normal (n=78)		Fragmented QRS (n=22)		t	p-value
	Mean	±SD	Mean	±SD		
MAP (Max)	119.78	12.35	119.23	13.62	0.18	0.856
MAP (Min)	74.37	13.59	75.91	15.58	-0.45	0.651
Diurnal Variation (S) - Heart Rate	16.40	4.56	17.33	6.72	-0.75	0.453

The percentages of normal dipping, extreme dipping, reverse dipping and non-dipping were 71.79%, 19.23%, 2.56%, and 6.41% in normal and 36.36%, 59.09%, 0.00%, and 4.55% in fQRS ECG, respectively. On the basis of dipping both groups were significantly different (Table 7).

**Table 7:** Qualitative changes in systolic blood pressure

Dipping	Normal (n=78)		Fragmented QRS (n=22)		Chi Sq.	p-value
	n	%	n	%		
Normal Dipping	56	71.79	8	36.36	13.77	0.003
Extreme Dipping	15	19.23	13	59.09		
Reverse Dipping	2	2.56	0	0.00		
Non-dipping	5	6.41	1	4.55		

The mean ejection fraction of Group 2 (fQRS; 46.65±13.11%) was found to be significantly lower as compared to Group 1 (normal QRS: 57.72±3.61%). 2D-Echo abnormalities were observed in a significantly higher proportion of Group 2 (fQRS; 58.8%) as compared to Group 1 (normal QRS; 2.2%) (Table 8).

**Table 8:** 2D-Echocardiography comparison between normal and fQRS subgroups EF: ejection fraction (left ventricular)

SN	Characteristics	Normal (n=78)	Fragmented QRS (n=22)	Statistical Significance
1.	Mean EF±SD (%)	57.72±3.61	46.65±13.11	t=4.323; p<0.001
2.	2D-Echo abnormalities	1 (2.2%)	10 (58.8%)	χ^2^=27.640; <0.001

Table 9 shows the linear regression analysis to assess the potential association between different parameters and fQRS ECG. The HR Peak DT, HR Peak NT, HR Min DT, HR Min NT, SBP Peak DT, SBP Min DT, DBP Peak DT, DBP Peak NT, DBP Av NT, DBP Min NT and diurnal variation(s) were significantly associated with fQRS.

**Table 9:** Linear regression analysis to assess the potential association between different parameters and fragmented QRS ECG HR: heart rate; DT: day time; NT: night time; AV: average; min: minimum; SBP: systolic blood pressure; DBP: diastolic blood pressure; MAP: mean arterial pressure; BMI: body mass index; BS: blood sugar; HbA1c: glycated haemoglobin; VLDL: very low density lipoprotein; LDL: low density lipoprotein; HDL: high density lipoprotein

	B	Std. Error	Beta	95% CI		p-value
				Lower	Upper	
HR Peak DT	0.01	0.01	0.52	0.00	0.02	0.039
HR Peak NT	-0.01	0.01	-0.49	-0.02	0.00	0.022
HR Av DT	0.01	0.01	0.48	0.00	0.03	0.081
HR Av NT	-0.01	0.01	-0.39	-0.02	0.00	0.051
HR Min DT	-0.02	0.01	-1.00	-0.03	-0.01	0.001
HR Min NT	0.02	0.01	1.09	0.01	0.03	0.002
SBP Peak DT	-0.01	0.00	-0.38	-0.02	0.00	0.037
SBP Peak NT	0.00	0.01	-0.07	-0.02	0.01	0.736
SBP Av DT	0.01	0.01	0.33	0.00	0.02	0.095
SBP Av NT	0.00	0.01	0.07	-0.01	0.02	0.740
SBP Min DT	-0.02	0.00	-0.77	-0.02	-0.01	0.000
SBP Min NT	0.00	0.01	-0.08	-0.01	0.01	0.655
DBP Peak DT	0.02	0.01	0.41	0.01	0.03	0.002
DBP Peak NT	-0.02	0.01	-0.46	-0.04	-0.01	0.000
DBP Av DT	0.02	0.01	0.39	0.00	0.04	0.124
DBP Av NT	0.01	0.01	0.34	0.00	0.03	0.040
DBP Min DT	0.00	0.01	0.10	-0.01	0.02	0.671
DBP Min NT	-0.01	0.01	-0.34	-0.03	0.00	0.040
MAP(Max)	0.00	0.00	-0.06	-0.01	0.00	0.497
MAP(Min)	0.01	0.00	0.17	0.00	0.01	0.054
Diurnal Variation(s)	0.05	0.01	0.66	0.03	0.08	0.000
Haemoglobin	0.02	0.02	0.10	-0.01	0.05	0.145
Total Leukocyte Count	0.00	0.00	-0.13	0.00	0.00	0.220
Platelets	0.06	0.04	0.11	-0.01	0.14	0.087
Height	0.01	0.01	0.14	-0.01	0.02	0.301
Weight	0.00	0.01	-0.09	-0.02	0.01	0.546
BMI	0.05	0.02	0.36	0.01	0.10	0.023
Waist Circumference	-0.02	0.00	-0.39	-0.03	-0.01	0.000
BS Fasting	0.01	0.00	0.65	0.00	0.01	0.000
BS Post-prandial	0.00	0.00	-0.10	0.00	0.00	0.477
Hb1AC	-0.14	0.03	-0.61	-0.21	-0.07	0.000
Serum Cholesterol	0.00	0.00	-0.42	0.00	0.00	0.000
Serum Triglyceride	0.00	0.00	0.18	0.00	0.00	0.114
VLDL	0.00	0.00	-0.12	-0.01	0.00	0.153
LDL	0.00	0.00	-0.08	0.00	0.00	0.350
HDL	0.00	0.00	-0.04	-0.01	0.01	0.661
Urea	0.00	0.00	0.21	0.00	0.01	0.044
Serum Creatinine	-0.26	0.08	-0.28	-0.42	-0.11	0.001

## Discussion

Individuals having metabolic derangements are at high cardiovascular risk (CVR) and the presence of multiple metabolic disorders increases this risk further. The presence of a cluster of these metabolic disorders namely obesity along with insulin resistance (diabetes), hypertension and dyslipidemia is termed as MetS. MetS is found to be associated with deterioration of the LV systolic and diastolic functions. One of the factors for this impairment is myocardial fibrosis. fQRS complexes are found to be associated with myocardial fibrosis [20]. Since treatment for myocardial fibrosis centres around addressing the underlying disease, it is important to treat MetS which can limit the worsening go cardiac functions. Hypertension is present in almost 80% of patients with MetS and pressure overload, induced by hypertension, results in extensive cardiac fibrosis; therefore, hypertension by BPV and fQRS is important to be studied from both preventive as well as early management points of view [21].

BPV as a confounding factor in CVD occurrence, severity and complication in the backdrop of metabolic disease has been extensively studied [22,23]. Considering fQRS as an indicator of complicated MetS with an increased risk of cardiac events, it is essential that the role of BPV in MetS patients should be interpreted in context with fQRS status which was the basis of our research. In our study amongst MetS inductees, fQRS was observed in 22% of patients while the remaining 78% had normal QRS, which is similar to a study by Oner [20] et al. that reported MetS prevalence of fQRS to be 26.1%. They also reported a higher prevalence of diabetes and hypertension along with higher glycemic levels, obesity parameters and hemodynamic parameters in MetS, a similar pattern seen in our study.

fQRS prevalence in hypertensives and diabetics shows a close relationship as in a study by Bekar et al. [24] and Yagi et al. [25] (52.2% and 36%). Yagi et al. also reported fQRS patients having higher age, waist circumference, heart rate, diabetes and MetS (51% vs. 30%), hypertension, total cholesterol and HbA1c.

In our study, fQRS patients were significantly associated with T2DM and CAD. The prevalence of hypertension was also higher among fQRS. The relationship between hypertension and T2DM in MetS does not require much elaboration as they are component factors of MetS and should not be viewed as a comorbid condition. However, the presence of both factors has been associated with CVR factors of their own.

Kanga [26] compared the heart rate variability (HRV) of 121 males with MetS and 131 male controls, 58 females with MetS and 191 control females. They observed men and women with MetS had lower total power and high-frequency (HF) power of HRV than controls whether supine or upright while men and women with MetS had lower upright low-frequency power of HRV than controls. After adjustment for age, smoking habits, alcohol intake, height, HR and breathing frequency, only the differences in upright total power and HF power of HRV between women with MetS and control women remained significant.

In the present study, fQRS MetS patients had significantly higher HRs (Peak Daytime, Av. Daytime and Av. Night time value). Systolic BP (Peak Day and Night, Av. Day and Night and Min. Night time values) and Diastolic BP (Peak, Av. and Min. values both day and night time). The association of fQRS with a diurnal variation of MAP was not found to be significant. The majority of fQRS MetS patients had been found to be extreme dippers (64.7%) while the majority of normal QRS MetS patients were found to be dippers. Reverse dippers were also higher among fQRS MetS as compared to MetS normal QR patients (5.9% vs. 4.3%). There are limited studies reporting any distinction in the BPV pattern of MetS patients. Ukkola et al. [27] who had enrolled 1770 non-diabetic untreated patients had reported non-dipping to be significantly associated with MetS. Felisbino-Mendes et al. [28] had reported pulse pressure (PP) (daytime, night time, av. 24h) was significantly elevated in cases of hypertriglyceridemia, dyslipidemia, overweight, and obese.

Age-adjusted MS was associated with higher CVR (OR = 4.5 and 3.6), 24-h PP (OR = 2.3 and 4.7), and daytime PP (OR = 2.2 and 4.6). They concluded that MS was highly prevalent and correlated with altered 24-hour BP parameters.

Taghia et al. [29] analysed the complete data on ABPM in patients with diabetes. They reported that approximately 54.2% of participants had non-dipping nocturnal patterns and 28.6% were risers. Non-dipping nocturnal BP was associated with CVD, neuropathy, and retinopathy.

Marcus et al. [1] established an association between MetS and BPV in a longitudinal study; they found that a one-year intervention resulted in weight reduction as well as a reduction in BPV. These findings establish that modulation of CVR through appropriate interventions has a direct impact on the BPV. In the present study, we could not carry out a longitudinal study but a cross-sectional study found that patients with higher CVR (fQRS) have a significantly different BPV pattern as compared to those without fQRS.

The findings of the present study showed that in an otherwise matched MetS population having no significant difference with respect to traditional biochemical markers (fasting blood sugar, postprandial blood sugar, Hb1c, serum lipids, serum creatinine) that are differentiated only by QRS fragmentation vis-à-vis ejection fraction and 2D Echo abnormalities. BPV can be a useful, easy and non-invasive measure to determine the risk of cardiac events.

Limitations

The present study included 100 patients of MetS in whom ABPM was done and subsequently correlated with fQRS. Although results are promising a larger sample size would go a long way to increase the power of the study. The presence of fQRS is more sensitive than Q wave on ECG to identify myocardial scar, yet it is less specific. A combination of fQRS with Q wave in a 12-lead ECG shows a 74% sensitivity and 92% specificity [30]. Thus, a combined assessment with both entities would have further increased the weight of the study. Also, the patients could have been followed up, to assess if the fQRS changes/resolution occurred with control of BP and lifestyle modifications.

## Conclusions

Ambulatory blood pressure is a useful, easy and non-invasive measure to determine the difference in BPV pattern in patients of MetS with high CVR (fQRS) as compared to those with normal QRS. fQRS is an important indicator of deteriorating cardiac patients and can be used to screen potential patients of cardiovascular insult who otherwise do not comply with 24-hour monitoring. Thus, early suspicion and tailored assessment would go a long way in reducing the financial burden, resources and extensive testing thereby reducing overall morbidity and mortality.
